# Access to innovative cancer medicines in a middle-income country - the case of Mexico

**DOI:** 10.1186/s40545-018-0153-y

**Published:** 2018-10-24

**Authors:** Daniela Moye-Holz, Rene Soria Saucedo, Jitse P van Dijk, Sijmen A Reijneveld, Hans V Hogerzeil

**Affiliations:** 1 Department of Community and Occupational Medicine, University Medical Center Groningen, University of Groningen, Hanzeplein 1, 9713 GZ Groningen, The Netherlands; 2 Boston University School of Public Health, Boston, USA

**Keywords:** Access, Drug utilization, Essential cancer medicines, Mexico, Insurance schemes access, Regional access

## Abstract

**Background:**

Cancer has become the third cause of death in Mexico. Treatment for cancer is often complex and lengthy. New and better medicines enter the market at high prices, which may limit access. Like most Latin American countries, Mexico has an essential cancer medicines list that includes innovative medicines. Their accessibility and use in the public sector remains unknown. Therefore, we describe the use, as a proxy of access, of innovative and essential cancer medicines in the public sector in Mexico, by insurance institution, and by five regions between 2010 to 2016.

**Methods:**

We used drug utilization research methods to assess the use of eight patented cancer medicines. Through the national transparency platform, we obtained data on the quantities of these medicines used in all public health facilities and social health insurance institutions and recalculated those figures into defined daily dose (DDD) per 1000 population per year.

**Results:**

Overall, the use of all medicines increased over the years, especially for trastuzumab, rituximab and imatinib. The use of innovative medicines was higher per population covered in social health insurance institutions than in governmental facilities. Throughout the study period, the Central region (including Mexico City) has used more medicines per population than the other regions.

**Conclusions:**

The use and access of some essential innovative cancer medicines has increased over the years, but remains unequal across insurance schemes and regions. Particularly, the Ministry of Health Insurance scheme and Northern and Western regions in the country would benefit from additional efforts to increase access to essential cancer medicines.

## Background

Cancer has become a leading cause of disability and mortality in the world, particularly in low and middle-income countries (LMIC) [1–3]. Such health care systems are not yet prepared to handle this burden [4]. In 2013, 12.8% of all deaths in Mexico were due to cancer [5]. Although Mexico has introduced specific health policies to tackle non-communicable diseases, like tobacco control, obesity control, and breast cancer screening, cancer remains the third leading cause of death in the country [5]. Like other Latin American (LATAM) countries, Mexico has invested significant resources to enhance preventive efforts - as many cancer cases are diagnosed at advanced stages - which typically have poor prognosis and high mortality [5]. Yet, factors such as lack of awareness on the importance of screening, poor distribution of screening programs, delays in pathology assessment and referrals, poverty, geographic barriers, lack of access to healthcare systems, financial barriers and stigma related to cancer have negatively impacted the improvement of cancer treatments and its outcomes [1, 4–7]. In addition, cancer cases diagnosed at later stages of the disease consume more resources, as treatments tend to be more complex [1, 5, 8].

In Mexico, the public sector provides most of the cancer care including cancer medicines [9]. This sector consists of five different social health insurance (SHI) institutions, each with their independent facilities and managerial styles, responsible for providing health coverage and care to the formal sector (employees and their families). The Mexican Social Security Institute (IMSS) is the main SHI institution, providing coverage to employees of private companies, approximately 46% of the population. The Institute for Social Security and Services for State Workers (ISSSTE) provides coverage to state employees, approximately 10% of the population. The National Defense Ministry (SEDENA), the Navy Ministry (SEMAR) and the National Oil Company (PEMEX) provide coverage to their employees, approximately 2% of the population [10]. These institutions cover cancer treatment according to their own guidelines, policies and medicine formularies. The population without SHI (roughly 42%) can receive healthcare at the Ministry of Health (MoH) facilities; each facility has its own policies and managerial style. Most of this population is affiliated to the People’s Health Insurance (Seguro Popular de Salud, SPS), which is a governmental insurance that reimburses health institutions according to a catalogue of interventions [11]. SPS covers all child cancer types and some of the most prevalent adult types, following its own guidelines and protocols. The MoH facilities have a list of selected medicines based on the national formulary and/or according to the list of medicines covered by SPS as described in its catalogue of interventions, which is also based on the national formulary [11–13].

The innovation field for cancer medicines is growing [14, 15]. New and better medicines are coming into the market, forcing constant updates of treatment protocols and formularies. Yet, most of the time, the high prices tagged to these innovations keep newer treatments unaffordable for individual patients [4] and burdensome for health systems, thus limiting patient’s access to new cancer medicines [16, 17]. As a result, prices rather than efficacy become a decisive factor for inclusion of these medicines in national or institutional formularies and ultimately, for reimbursement [1, 18].

Access to new cancer medicines is a challenge in all LMIC [19]. Most LATAM countries – including Mexico – utilize essential medicines lists for procurement purposes [1], which should guarantee proper access in health centers [20]. However, differences in access to these medicines across insurance schemes and country regions is not well known [21]. For example, some European countries and Australia have performed drug utilization studies to describe the availability and utilization of these medicines across regions and countries. These methodologies can inform about the distribution and the uptake of resources (e.g. cancer medicines); but these methodologies have rarely been used in middle-income countries (MIC), including Mexico [19, 22–24]. Therefore, this study describes the use of patented cancer medicines in the Mexican public sector, comparing five SHI schemes and the MoH in five geographic regions, and changes between 2010 and 2016.

## Methods

### Cancer medicines studied

We selected medicines based on the following criteria: inclusion in the national formulary, coverage by the SHI institutional lists, coverage by SPS, inclusion in the national clinical guidelines and SPS treatment guidelines (protocols), patent protection in Mexico until after 2017. We narrowed our selection of medicines based on the criterion that medicines should have gone through price negotiations every year from 2010 to 2016 by the Mexican Coordinating Commission for Negotiation of Prices of Medicines (CCNPMIS) [25]). The CCNPMIS is a commission that negotiates directly with pharmaceutical companies the public procurement prices applicable for the public sector only. The CCNPMIS determines which medicines will be considered for negotiations taking into consideration their relevance, estimated demand and procurement volume [25, 26]. The latter characteristics indicate that these medicines are considered both innovative and essential in Mexico, and that they could have been procured in the public sector during that period of time. Following these criteria, we selected the following medicines: bevacizumab, dasatinib, imatinib, nilotinib, rituximab, sorafenib, sunitinib, and trastuzumab. Some of these medicines (nilotinib and sorafenib) are not covered by SPS; furthermore, some of these are covered by SPS only for children or could be covered in case of disease progression (bevacizumab, dasatinib, sunitinib). We decided to include them because they had been negotiated by the CCNPMIS and included in the national clinical guidelines.

This range of medicines reflects some of the main cancers prevalent in Mexico. Imatinib, dasatinib and nilotinib are indicated for leukemia [27], the most prevalent cancer in children in Mexico [28]. Rituximab is indicated for non-Hodgkin lymphoma (NHL) treatment in addition to leukemia. Trastuzumab is indicated for breast cancer, one of the most common causes of dead among women in Mexico [29]. Bevacizumab, one of the most frequently used anti-cancer medicines worldwide, is indicated as a first line treatment for advanced colorectal cancer [30, 31], which has an increasing incidence in Mexico [32]. Sorafenib and sunitinib are both indicated for renal cancer; sunitinib is covered by SPS only for children.

### Measures and procedure

Procurement data (volume and value) from the public sector were retrieved through the National Transparency Platform (PNT) [33]. Procurement data from all possible public health institutions providing cancer care in the country were obtained from the various institutions that provide this type of care (see Table 1).

**Table 1:** Public Health Institutions and Social Health Insurance Institutions providing cancer care in Mexico

Ministry of Health (MoH)	
Each states’ Ministry of health and/or state health services (SESA)	
Mexican Social Security Institute (IMSS)	
Institute for Social Security and Services for State Workers (ISSSTE)	
National Defense Ministry (SEDENA)	
Navy Ministry (SEMAR)	
National Oil Company (PEMEX)	
National Institute of Cancerology (INCAN)	
National Nutrition Institute (INNSZ)	
National Institute of Pediatrics (INP)	
Federal Hospitals	
Regional high specialty hospitals (HRAE)	

We used the defined daily dose (DDD) as the unit of measurement of use, in order to standardize and add the quantities procured and allow for proper comparisons. Because the WHO has not yet defined DDDs for most cancer medicines we used DDD values as reported by the German national Anatomic-Therapeutic-Chemical classification [34].

To measure the use or utilization rates of these medicines among the five SHI institutions and the MoH, population numbers affiliated to each type of health provider were used, as reported by the National Institute of Statistics and Geography (INEGI) [10]. To measure access among geographic regions, state population affiliated to SPS data were used, as reported by the SPS [35].

### Data analysis

**Fig. 1:**
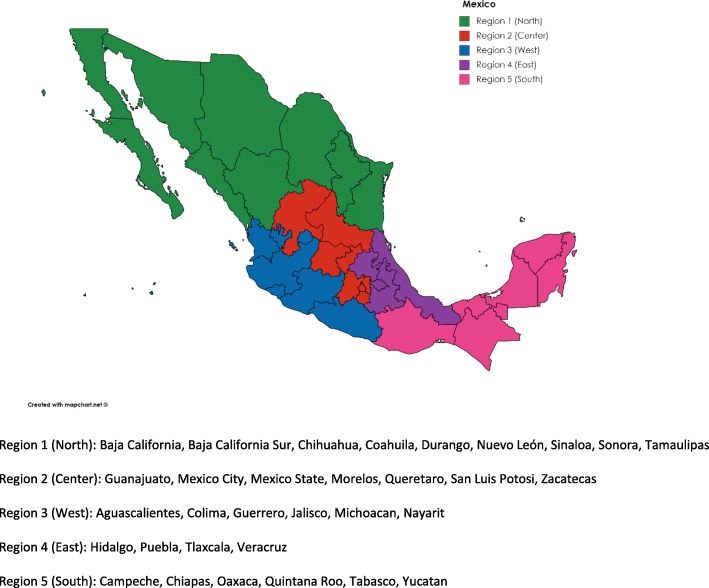
Mexico – the five regions of study. Region 1 (North): Baja California, Baja California Sur, Chihuahua, Coahuila, Durango, Nuevo León, Sinaloa, Sonora, Tamaulipas. Region 2 (Center): Mexico City, Mexico State, Guanajuato, Morelos, Querétaro, San Luis Potosí, Zacatecas. Region 3 (West): Aguascalientes, Colima, Guerrero, Jalisco, Michoacan, Nayarit. Region 4 (East): Hidalgo, Puebla, Tlaxcala, Veracruz. Region 5 (South): Campeche, Chiapas, Oaxaca, Quintana Roo, Tabasco, Yucatan.

Standard drug utilization research methods were used [36]. First, we analyzed the data on the eight medicines from 2010 to 2016 separately, in order to assess their individual use rates in DDDs/1000 inhabitants. Secondly, we expressed differences in access according to health insurance schemes as DDDs/1000 persons covered. We also compared access between the regions for MoH channels only, regrouping 32 states into five regions [37] (Fig. 1) and expressing overall access to all products together as total DDDs/1000 inhabitants per region. We regrouped the country into 5 geographical regions following our own discretion into northern, center, western, eastern and southern regions, derived from the Ministry of Education’s classification [38].

## Results

### Differences in access to innovative cancer medicines in Mexico

**Fig. 2:**
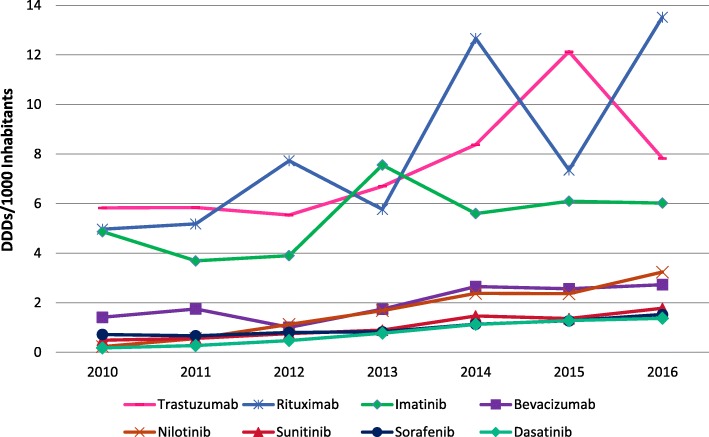
Annual quantities of eight essential cancer medicines procured in the public sector (SHI institutions and MoH) in Mexico (2010–2016)

Figure 2 shows quantities procured of the eight selected cancer medicines from 2010 to 2016 in public facilities in Mexico. Overall, the annual quantities procured have increased for all medicines under study. The most commonly used medicines were imatinib, rituximab and trastuzumab. The quantities of rituximab and imatinib have remained high throughout the years, while trastuzumab shows a considerable increase since 2012 and a decrease between 2015 and 2016. The quantities of bevacizumab, dasatinib, nilotinib, sorafenib and sunitinib have remained steadily increasing, but in much lower quantities.

### Access to innovative cancer medicines in the public sector

**Fig. 3:**
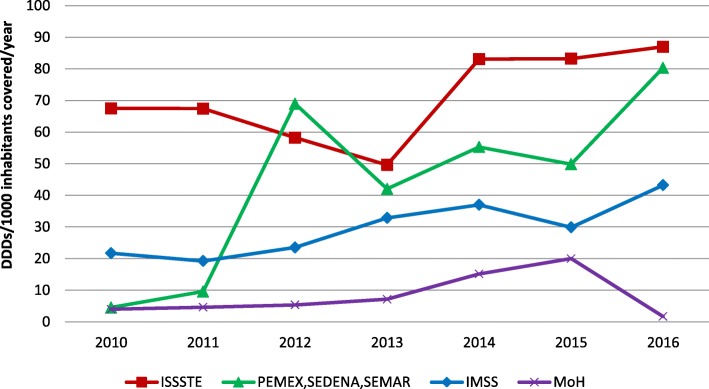
Total annual quantities of eight essential cancer medicines, procured in the Mexican public sector (SHI institutions and MoH) per insurance scheme (2010–2016). *1000 inhabitants covered per SHI institution and by the MoH/SPS; for abbreviations see Table 1

Figure 3 shows that quantities of innovative cancer medicines procured by different SHI institutions and the MoH have increased over the years (especially since 2013). The quantities of medicines procured by IMSS and the MoH have remained lower than by other SHI institutions in the period of study. Among SHI institutions, ISSSTE has procured larger quantities than all other institutions. IMSS procured the largest volume of medicines, but when adjusted to quantities procured per population covered (approximately 50%), it has the lowest rates among the five SHI institutions. The SHI for the oil company (PEMEX), the army (SEDENA), and the navy (SEMAR) have increased their use the most since 2011, and have had constantly higher quantities per population covered than IMSS and MoH.

### Regional quantities of innovative cancer medicines

**Fig. 4:**
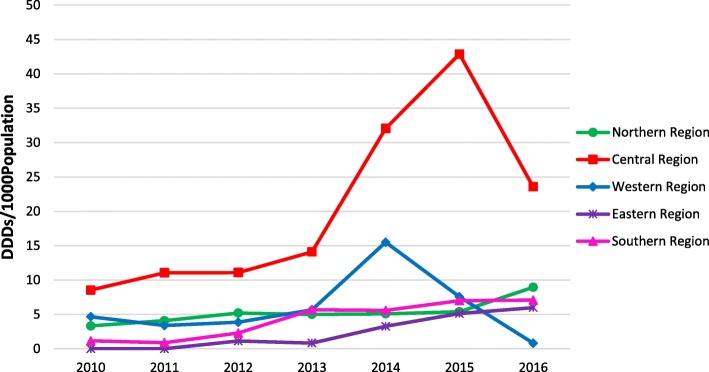
Total annual quantities of eight essential cancer medicines, purchased in MoH facilities, per region (2010–2016). *1000 inhabitants covered by SPS per region

Figure 4 shows that quantities of innovative cancer medicines procured by the MoH remained lower in the northern, western, eastern and southern regions than in the central region (including Mexico City). In all regions, quantities have remained largely the same from 2010 to 2013. From 2013 to 2015, most regions experienced an increase, particularly the central region. However, the western and the southern region experienced a decrease in the quantities of medicines procured since 2014 and 2015 respectively.

## Discussion

To our knowledge, this is the first study to describe the use of cancer medicines across the Mexican public sector. Reporting use of medicines provides a proxy measure of access to medicines and allows for comparisons between different settings (e.g. insurance schemes, geographical regions). First, the amount of DDDs of rituximab, imatinib and trastuzumab account for more than 70% of the total of DDDs of all procured medicines under study. Second, SHI institutions provide larger quantities per insured population than the MoH. Third, the central region (including Mexico city) reports, on average, a constantly higher use of cancer medicines than the other regions, which continued to have a low level of use.

### Access barriers to individual medicines

All medicines under study showed an increase in quantities procured throughout 2010–2016. For most of them, this increase was slow and only for some medicines, in particular imatinib, rituximab and trastuzumab, the increase was larger. These three medicines were covered by all SHI institutions and by the SPS. These medicines have demonstrated improved health outcomes [21], which has been recognized by the WHO and justifies their inclusion in the WHO-EML since 2015 [21]. However, only SPS does not fully cover dasatinib, nilotinib, bevacizumab, sunitinib and sorafenib [27]. Low accessibility of effective innovative medicines could limit adequate cancer care [16], particularly for the most vulnerable populations with colorectal and renal cancer.

Use and access to new cancer medicines is low in Mexico, with levels similar to those reported from other developing regions such as, for example, Africa, South-East Asia and Latin America [39, 40]. Studies performed on the use of some innovative medicines in the USA, Russia, Turkey, Brazil and Mexico [41, 42], have reported that barriers to access and use of innovative cancer medicines link to limited coverage by public insurance schemes, inclusion in the EML, availability of the medicine at the facilities, and updated clinical guidelines. The lack of availability in the public sector has pushed patients in Mexico, Russia and Brazil to get these medicines in the private sector and pay out of pocket [41, 42].

### Access barriers by health coverage

We found large differences in use linked to the type of health coverage. For example, all eight medicines studied were covered by all SHI institutions (IMSS, ISSSTE, PEMEX, SEDENA, SEMAR) but only three were covered by the governmental SPS for children and adults (imatinib, rituximab, trastuzumab), and another two medicines where covered by SPS only for children (dasatinib and sunitinib), therefore limiting use of the other medicines in the MoH facilities [27]. Other studies have consistently reported higher availability and accessibility rates for essential medicines at the IMSS than other institutions in the public sector [43–47]. Previous research reported that MoH and IMSS are the largest providers of cancer care in the country [9]; despite this, access to the medicines of study at these two institutions [32] remained lower than at the other SHI institutions when expressed as quantities used per population insured. High prices of medicines, financial barriers, budget constraints, and the lack of prioritization of cancer care could explain the differences among institutions, highlighting the inequalities in access to innovative medicines and health care [16, 32]. This is worrisome as it could indicate that over 80% of the population experiences barriers to innovative medicines that could provide them with better outcomes of their treatment against cancer.

### Access barriers by geographic location

We found regional variations in the use of the studied medicines, in line with previous findings from other countries [24, 31, 48–50]. These variations could be due to differences in the burden of disease, budget and resource allocation, purchasing power, differences in capacity within the health care system and disease priorities [16, 36, 51]. Like other LATAM countries, Mexico concentrates resources and health care in big cities (e.g. Mexico City, Monterrey and Guadalajara in the central, northern and western region respectively). Such a policy generates an “overwhelming influx of patients” [1, 52], which may have led to the relatively large increase in provision in recent years. Based on the number of hospital discharges, these three regions report high proportions of cancer patients attended by the MoH [9, 53]. Yet, use is far greater in the central region than in all other regions. In addition, distance to health facilities and traveling costs prevent patients from seeking health care and getting treatment [32, 54–56]. Thus, decentralization of health care is needed to bring treatment closer to patients, and improve access and health outcomes in regions currently lagging behind.

We also found a decrease in the quantities of medicines procured at MoH facilities in 2016, particularly in the central and western regions, largely explained by decreased quantities of trastuzumab. Trastuzumab had experienced constant increases in use particularly from 2012 to 2015 and had a sudden drop in 2016 (Fig. 2). This finding is unexpected, since Mexico has invested efforts in the control of breast cancer [29, 57]. The reasons for this decrease and the possible barriers that MoH facilities face when procuring trastuzumab need to be further explored.

Research in the US, Australia, China, Canada and Sweden suggest that geographic variations in access to innovative medicines [23, 24, 31, 48–50] could be explained by differences in coverage, insurance guidelines and management of the disease, professional disagreement and prescription preferences, budget issues and local policies. Heterogeneity in the burden of disease can also influence allocation of resources to a specific type of medicines [16, 23]. In the case of China, regional differences were also attributable differences in access to health care [48].

### Strengths and limitations

The strength of this study lies in the collection of data from all public health institutions in the whole country, which allowed for a comprehensive overview on procurement and use of the selected medicines and for presenting differences between geographic regions and insurances schemes. Potential limitations include that we were unable to retrieve data from some states (e.g. Michoacan) while some other states provided incomplete data (e.g. Nayarit, Quintana Roo, Nuevo Leon, Guerrero), particularly before 2014. Our regional results (particularly northern, western and southern regions) may therefore underestimate the real quantities. Another limitation is that we did not take into account any regional variations in cancer burden, which could affect the quantities of medicines needed. Furthermore, this study focused on a selected number of innovative cancer medicines and does not account for a whole treatment scheme and does not differentiate use according to burden of each disease. Further research should focus on complete treatment schemes and weigh the use of these medicines against the burden of diseases (e.g. using mortality, incidence and/or morbidity data).

### Implications

At the organizational level, use of medicines through IMSS and the MoH was lower than through other SHI institutions. Since IMSS and the MoH together cover most of the population, a more detailed analysis is needed to identify the barriers preventing adequate use and access to cancer essential medicines. Differences between regions continue to reflect a concentration of resources in the center of the country and limited infrastructure to manage specialized health care needs in the rest of the regions.

Previous research on access to innovative cancer medicines in LMIC has focused on whether these are included in national EMLs [20, 58]. Further research should now focus on use and actual access to comprehensive treatment schemes of the most prevalent types of cancer [20], in order to inform stakeholders and policy makers on the current situation and identify potential barriers to be addressed. Public health institutions and the government need to reflect on how resources can be allocated more equally and efficiently to ensure universal access to the most cost-effective level of care. Improving access and use of innovative treatments of which the effectiveness, safety and cost-effectiveness have been established, will provide better quality of cancer care, better health outcomes and fewer deaths due to cancer [49, 51]. The government should monitor the needs for these medicines along with their actual use and access to guarantee the best level of care. Efforts on improving access to cancer medicines need to go along with better access to screening, prevention and other types of treatment.

## Conclusions

Over the last 6 years, the use of eight innovative essential cancer medicines has increased in Mexico, particularly of imatinib, rituximab and trastuzumab. The use of five other essential cancer medicines has remained low due to insufficient insurance coverage. Regional differences in the use of innovative cancer medicines highlight inequalities in access to cancer care. Providing access to essential innovative cancer medicines remains a challenge in Mexico. Further decentralization of cancer care is warranted to improve equitable access and use of effective and affordable cancer treatments.

## Abbreviations

CCNPMIS: Coordinating Commission for Negotiation of Prices of Medicines
CONAPO: Population National Council
DDD: Defined daily dose
EML: Essential Medicines List
HRAE: Regional high specialty hospitals
IMSS: Mexican Social Security Institute (Insituto Mexicano del Seguro Social)
INCAN: National Institute of Cancerology
INEGI: National Institute of Statistics and Geography
INNSZ: National Nutrition Institute
INP: National Institute of Pediatrics
ISSSTE: Institute for Social Security and Services for State Workers (Instituto de Seguridad y Servicios Sociales de los Trabajadores del Estado)
LATAM: Latin America
LMIC: Low- and Middle-Income countries
MIC: Middle Income Countries
MoH: Ministry of Health
NHL: Non-Hodgkin Lymphoma
PEMEX: National Oil Company (Petróleos Mexicanos)
PNT: National Transparency Platform
SEDENA: National Defense Ministry (Secretaría de la Defensa Nacional)
SEMAR: Navy Ministry (Secretaría de la Marina)
SESA: State health services
SPS: People’s Health Insurance (Seguro Popular de Salud)
WHO: World Health Organization
